# Highly Sensitive Balloon-like Fiber Interferometer Based on Ethanol Coated for Temperature Measurement

**DOI:** 10.3390/s24113684

**Published:** 2024-06-06

**Authors:** Xin Ding, Qiao Lin, Shen Liu, Lianzhen Zhang, Nan Chen, Yuping Zhang, Yiping Wang

**Affiliations:** 1 Key Laboratory of Optoelectronic Devices and Systems of Ministry of Education, College of Physics and Optoelectronic Engineering, Shenzhen University, Shenzhen 518060, China; danielxin2019@126.com (X.D.); qiaolin3328@163.com (Q.L.); ypwang@szu.edu.cn (Y.W.); 2 Guangdong Laboratory of Artificial Intelligence and Digital Economy (SZ), Shenzhen 518107, China; 3 School of Intelligent Manufacturing, Shandong Polytechnic College, Jining 272067, China; zlzcnn@outlook.com; 4 School of Electrical Engineering and Automation, Nantong University, Nantong 226019, China; ntu_chennan@ntu.edu.cn; 5 SINOPEC Research Institute of Safety Engineering Co., Ltd., Qingdao 266000, China; zhangyuping1985@icloud.com

**Keywords:** balloon-like fiber interferometer, ethanol solution, temperature sensor

## Abstract

A highly sensitivity balloon-like fiber interferometer based on ethanol coating is presented in this paper. The Mach–Zehnder interferometer is formed by bending a single-mode fiber to a balloon-like structure and nested in the Teflon tube. Then, an ethanol solution was filled into the tube of the balloon-like fiber interferometer by the capillary effect. Due to the high sensitivity of the refractive index (RI) of ethanol solutions to temperature, when the external temperature varies, the optical path difference changes. The change in temperature can be detected by the shift in the interference spectrum. Limited by the size of the balloon-like structure, three kinds of these structures with different sensitive lengths were prepared to select the best parameters. The sensitive lengths were 10, 15 and 20 mm, respectively, and the RI detection performance of each structure in 10~26% NaCl solutions was investigated experimentally. The results show that when the sensitive length is 20 mm, the RI sensitivity of the sensor is the highest, which is 212.88 nm/RIU. Ultimately, the sensitive length filled with ethanol is 20 mm. The experimental results show that the temperature sensitivity of the structure is 1.145 nm/°C in the range of 28.1 °C~35 °C, which is 10.3 times higher than that of an unfilled balloon-like structure (0.111 nm/°C). The system has the advantages of low cost and easy fabrication, which can potentially be used in high-precision temperature monitoring processes.

## 1. Introduction

An accurate measurement of temperature is of great significance in promoting the development of the industry, and the use of optical fibers for temperature measurement can be traced back to the early 1970s when researchers found that the bending and elongation of optical fibers could have an impact on the optical signal, and therefore there was a subsequent use of the effect of temperature as a measurement. In the decades, fiber-optic temperature sensors have been extensively studied for their compactness, fast response, corrosion, and electromagnetic interference resistance. Currently, fiber-optic based temperature sensors (FOTSs) can be divided into four categories: fiber grating-type temperature sensors, interferometric FOTSs, surface plasmon resonance-type FOTSs, and distributed temperature sensors (DTS). Fiber grating temperature sensors include Fiber Bragg Gratings (FBG) [1], Long Period Gratings (LPG) [2], etc. Fiber grating temperature sensors have a mature preparation process and a compact structure. However, the relatively low measurement sensitivity and temperature resolution (±1 °C) limits its further use. There are also some fiber grating sensors based on special fiber structures [3,4,5,6,7,8,9], including few-mode fibers, hollow-core fibers, photonic crystal fibers, multi-core fibers, etc., which can make up for the above shortcomings very well, but they require relatively complex preparation processes and are limited in mass production. DTS based on the principles of Raman, Rayleigh, or Brillouin scattering belong to distributed sensors [10,11,12]. In comparison, distributed sensors are more used for long-distance transmission such as oil field monitoring, which can be considered a non-active (light transmission type) sensor.

In the last few years, FOTSs based on interferometric principles, including Fabry–Pérot interferometers (FPI) [13,14,15], Mach–Zehnder interferometers (MZI) [16,17,18], Michaelson interferometers (MI) [19,20], and Sagnac interferometers (SI) [21,22,23], have become more and more popular because of the advantages of high sensitivity and the ability to maintain a high level of stability and reproducibility in harsh environments. Among them, the most favored structure for building various types of fiber-optic sensors is the MZI. To date, an extensive range of MZI-based FOTSs have been created by utilizing multiple fiber-optic topologies for precise design. Although these fiber-optic temperature sensors are able to provide an acceptable sensitivity, their drawbacks are obvious, including low mechanical strength, complex structure preparation processes, which usually require the use of expensive micro-nano processing equipment including femtosecond lasers, CO_2_ lasers [24,25,26], and high-precision fiber-optic tappers, and all of the above severely limit the use of such sensors in practical sensing systems. Therefore, the preparation of a simple and repeatable fiber-optic interferometer is becoming a hot research topic for many researchers. A novel idea is offered via creating mode interference by bending a single-mode fiber (SMF). Compared with common MZI structures (including SMS, tapered fibers, fiber misalignment fusion splicing, etc.), bent fibers are simple in structure, high in mechanical strength, and easy to fabricate. Due to the growing popularity of optical fiber communication networks worldwide, FOTSs based on bent optical fibers have been published, and the bending loss characteristics of optical fibers have also been extensively investigated [27,28,29,30,31,32,33,34]. In 2009, Ginu et al. [27] exposed a layer of material outside the cladding by removing the original coating layer, which was used to eliminate the whispering-gallery mode (WGM), thus effectively reducing the influence of other factors when performing temperature measurements. Peng et al. [28] used a nickel-plating process to metalize and encapsulate the bent optical fiber sensors and utilize monitoring temperature in harsh environments, with a temperature sensitivity of 0.023 dB/°C. Al-Janabi et al. [29] investigated the temperature effects of the original coating layer of the optical fiber, the polyvinyl alcohol (PVA) coating layer, and no coating layer on the optical fiber macro-bending sensors, and found that the temperature sensor based on the PVA coating layer had the highest sensitivity, with a measurement range of 30~55 °C. Nam et al. [30] reported a thinned high-temperature sensor based on the WGM excitation of the bending cladding, but the sensor’s temperature sensitivity was only 0.212 nm/°C. The above-mentioned bending fiber-optic sensors have unsatisfactory temperature detection performance, and so in this paper, the capillary effect is exploited to greatly simplify the preparation process of the sensors by filling the Teflon-covered outer cladding with a high temperature-sensitive material, ethanol, and at the same time further improving the temperature sensing performance of this structure.

This paper demonstrates a highly sensitive temperature sensor based on a balloon-like fiber structure, which achieves the modulation of cladding modes in an SMF based on the response of the refractive index of the outer coating (ethanol) to the temperature, thus enabling the detection of the external temperature. The balloon-like structure with a bending radius of 4 mm can effectively establish a modal interferometer based on propagating core modes and excited cladding modes. The refractive index (RI) was first experimented with by comparing the bending SMF stripped of three coating lengths (10 mm, 15 mm, and 20 mm), and it was finally determined that the RI detection sensitivity of the structure was higher when stripped of the coating layer of 20 mm. And then it was nested in a Teflon tube filled with ethanol solution. The sensor structure in this paper has been better in temperature sensing compared to the uncoated ethanol balloon-like structure, which can be attributed to the high thermo-optic coefficient of ethanol. The results show that the structure has a high sensitivity to temperature and good repeatability in the 28 °C to 35 °C range. Moreover, the proposed sensing system in this paper is low-cost, easy to fabricate, and has potential competitive advantages in the area of high-precision temperature measurement.

## 2. Sensing Structure and Operating Principle

**Figure 1:**
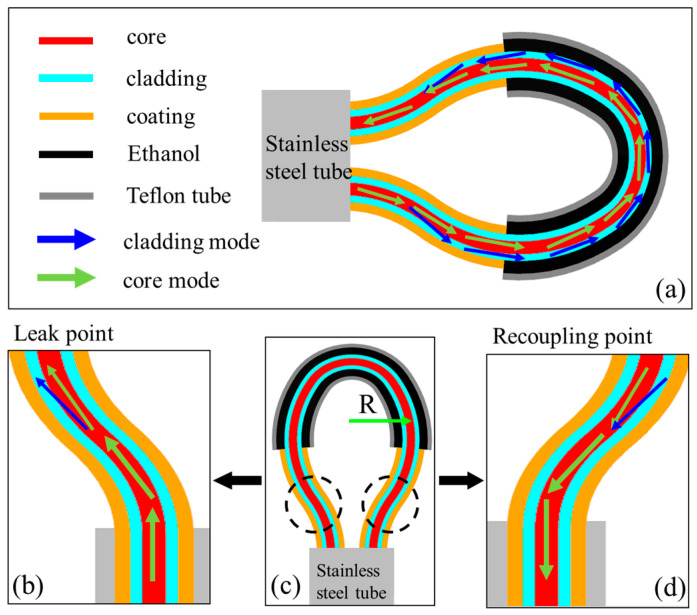
Schematic diagram of proposed structure. (**a**) Schematic diagram; (**b**) Enlarged diagram of leak point; (**c**) Overall structure diagram; (**d**) Enlarged diagram of recoupling points.

The schematic structure of the proposed balloon-like fiber-optic temperature sensor is shown in Figure 1a. A section of commercial SMF-28 (Corning, New York, NY, USA) with a stripped coating layer of approximately 20 mm is nested in a Teflon tube, which is then bent into a balloon shape using a stainless steel tube, followed by ethanol filling by the capillary effect, and finally encapsulated by curing using UV glue. The bending radius of the balloon-like structure is defined as R (Figure 1c). The working principle of this structure is based on the interference between the core modes and the excited cladding modes, which can be described as follows: the light from the light source enters the core of the optical fiber and is transmitted forward. When the light in the core enters the bending region (Figure 1b), part of the light will be unbound by the core and escape into the cladding, where the cladding modes are excited, and the cladding modes in the SMF are further coupled into the ethanol coating layer. The excited mode propagates through the cladding and when it reaches the coating/air boundary, part of the energy is reflected back to the core. Thus, when light propagates through the balloon-like structure, there is a periodic reflection between the core modes and the cladding modes, and a modal interferometer is formed due to the difference in the effective RI between the two modes. When light passes through the bending region, some of the cladding modes are recoupled back to the fiber core (Figure 1d). The intensity of light transmission through the balloon-like structure can be expressed as [33]

(1) $$I_{out}=I_{core}+I_{cladding}+2\sqrt{I_{core}I_{cladding}}cos\varphi$$

where $I_{cladding}$ and $I_{core}$ represent the intensities of the cladding and core modes, and φ denotes the phase difference between them, which can be expressed as

(2) $$\varphi =\frac{{2\pi ∆n}_{eff}L_{eff}}{\lambda }$$

where λ represents the wavelength of the light signal in free space, ${∆n}_{eff}$ refers to the effective RI difference between the cladding and core modes, and $L_{eff}$ is the effective bending length of the balloon-like area. An interference dip occurs at specific wavelengths when the condition of φ=(2m+1)π, m = 0, 1, 2,....

Combined with Equation (2), it can be seen that the resonant wavelength in the transmission spectrum of this structure can be expressed as

(3) $$\lambda_{m}=\frac{2{\Delta n}_{m,eff}L_{eff}}{2m+1}$$

By differentiating around Equation (3),

(4) $${d\lambda }_{m}=\frac{2}{2m+1}[d{\Delta n}_{m,eff}L_{eff}+{\Delta n}_{m,eff}dL_{eff}]$$

Because $L_{eff}$ remains the same, in the process of the RI measurement so $dL_{eff}=0$. The change in the resonant wavelength can be expressed as

(5) $${d\lambda }_{m}=\frac{\lambda_{m}}{{\Delta n}_{eff}}d{\Delta n}_{eff}$$

Among them, $d{\Delta n}_{eff}$ is the change in the effective RI difference between the core mode and the *m* cladding mode caused by the change in the external RI. As can be seen from Equation (5), when the excited cladding mode is a lower-order cladding mode, the interference spectrum will move towards the shortwave direction with the increase in the external RI, that is, $d{\Delta n}_{eff}<0$. However, when the excited cladding mode belongs to the higher order, the interference spectrum will move towards the long wave direction with the increase in the external RI, that is, $d{\Delta n}_{eff}>0$.

In this paper, the RI of ethanol on a balloon-like SMF surface decreases with the increase in ambient temperature, and the RI of ethanol is related to temperature [35] as follows:

(6) $$n_{1}=n_{0}-\alpha (T-T_{0})$$

where T represents the working temperature, and $T_{0}$ represents the initial temperature (20 °C); $n_{1}$ and $n_{0}$ represent RI at the temperature T and $T_{0}$, respectively. When $T_{0}$ is 20 °C, the RI of ethanol is 1.36048. α is the thermo-optic coefficient of ethanol, and the value is 4.45 × 10^−4^/°C, while the thermo-optic coefficient of SiO_2_ material, α, is 8.60 × 10^−6^/°C. It can be seen that the thermo-optic coefficient of the two materials is two orders of magnitude different.

The proposed modal interferometer based on the balloon-like structure in this paper can sense the external physical quantities in surroundings by detecting the shift in the resonant wavelength. The effective RI of the cladding modes and the effective length of the interferometer are altered by the changes in the surroundings accordingly. When the proposed structure is perturbed by the external temperature, the RI of the ethanol coated on the surface of the balloon-like structure undergoes corresponding changes, thus the RI of cladding modes is altered, and thus the phase difference value will change with the change in ${∆n}_{eff}$, which ultimately results in a wavelength shift in the transmission spectrum of the balloon-like interferometer, indicative of the temperature change. In our previous work [36], we have discussed the details of the characterization of a balloon-like structure and analyzed the transmission spectrum and spatial frequency domain of this structure with the SMF coating layer removed at different bending radii using the fast Fourier transform. It can be found that a bending radius of 4 mm yielded a significant interference dip with a good extinction ratio, making it the optimal value. Therefore, the use of this particular bending radius in our study is justified.

## 3. Sensor Fabrication Method and Experimental Setup

### 3.1. Balloon-like Fiber Sensor Fabrication

**Figure 2:**
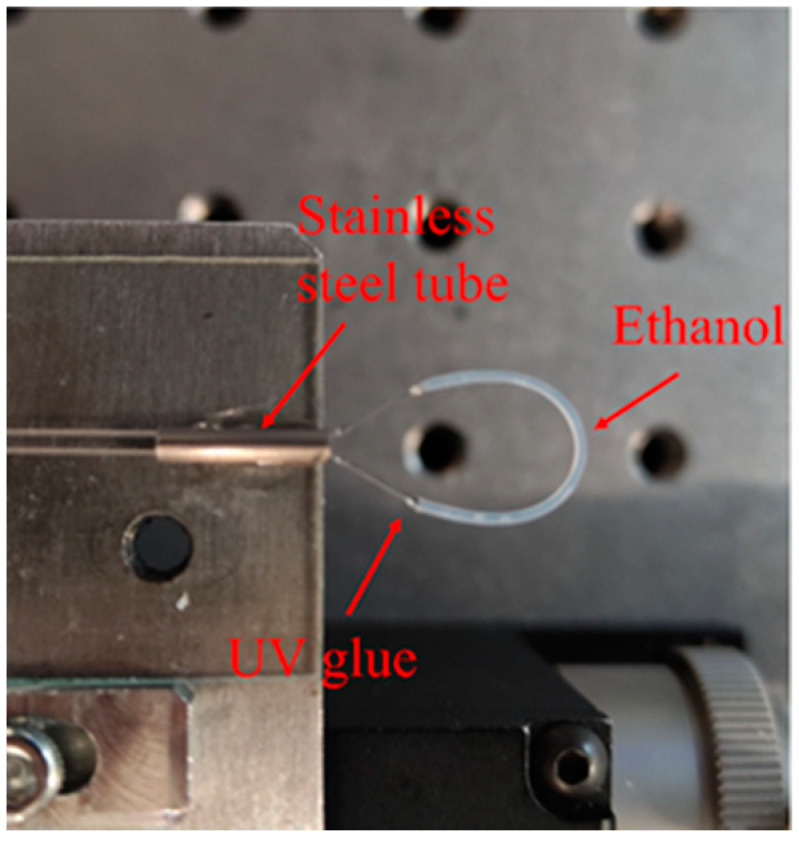
The balloon-like structure filling with ethanol.

The fabrication process of the balloon-like fiber sensor consisted of several steps. Firstly, the coating layer of the SMF was peeled off about 20 mm (sensing region) and then embedded in a Teflon tube, and it was made sure that the Teflon tube completely covered the uncoating area of the SMF. Here, the inner diameter and length of the Teflon tube are 0.3 mm and 25 mm. Secondly, the Teflon tube-wrapped SMF was bent into a balloon-like configuration using two micro-displacement platforms. Thirdly, ethanol solution was added to one end of the balloon-like structure using a dropper, and according to the capillary effect, the ethanol solution would gradually diffuse and fill the entire sensing area. Finally, UV glue was used at each end of the Teflon tube and then cured with a UV lamp to complete the encapsulation of the overall structure. The photograph of the prepared balloon-like fiber temperature sensor is provided in Figure 2.

**Figure 3:**
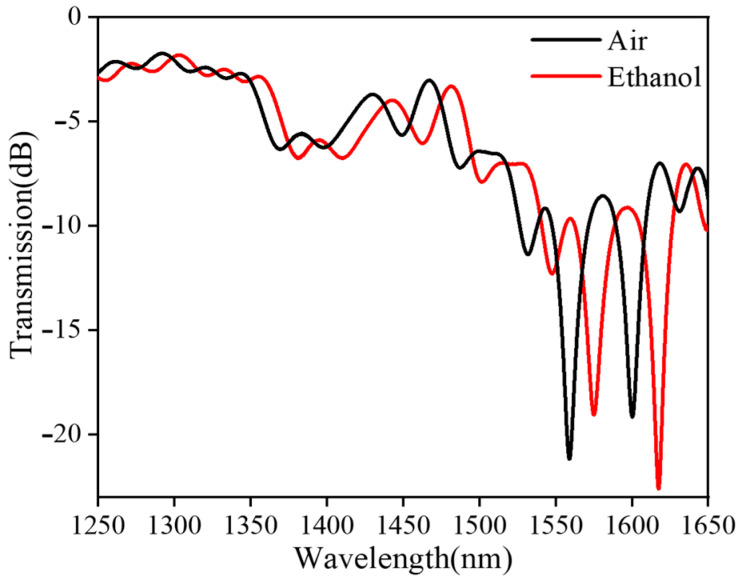
Transmission spectrum of sensor with/without ethanol.

The resonance depth of the transmission spectrum is mainly determined by the energy distribution between the cladding mode and core mode. When the bending radius is large, the energy coupled from the core mode to the cladding mode is too small, resulting in a lower resonance depth; when the bending radius is small, a relatively large amount of energy is transferred from the fiber core to the cladding, causing $I_{1}$ to be too small, resulting in a weakening of the resonant peak. When the energy of $I_{1}$ and $I_{2}$ is equal, the fiber optic interference structure has the highest contrast. The relationship between the bending radius and the resonance depth of the resonant peak has been detailed and explained in our previously published paper [37,38,39]. In Figure 3, we present a comparison of the transmission spectra of the balloon-like structure before and after filling with ethanol. In the absence of ethanol, the resonant wavelength dip undergoes a shift due to the higher RI of ethanol compared to air.

### 3.2. Experimental Setup for RI and Temperature Measurement

**Figure 4:**
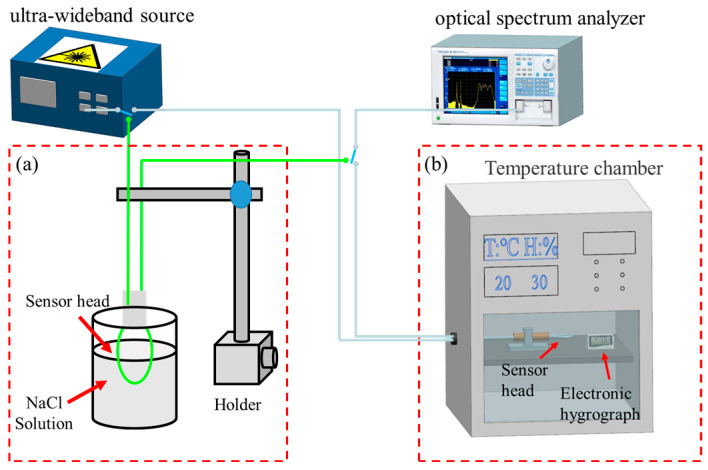
Experimental setup. (**a**) RI measurements; (**b**) temperature measurements.

The schematic diagram of the experimental setup for the RI measurement of the balloon-like structure proposed in this paper is depicted in Figure 4a. It comprises an ultra-wideband light source (UWS, Fiberer, operating in the wavelength range from 1250 to 1650 nm) and an optical spectrum analyzer (OSA, Yokogawa AQ6370D, Tokyo, Japan, operating wavelength from 600 nm to 1700 nm). Before the RI measurement experiment, it is necessary to be equipped with different RI solutions to be measured, and for the selection of different RI solutions, it is necessary to consider the viscosity, the range of RI changes, and the uniformity of RI distribution of the solutions to be measured. Ultimately, the RI solutions utilized in this experiment are different concentrations of NaCl solutions with concentrations of 10%, 14%, 18%, 22%, and 26%. A certain mass of NaCl solid was first weighed using a scale, followed by mixing with distilled water in different proportions [40]. The RI values of NaCl solutions detected by the Abbe refractometer (ATAGO, DR-A1 Plus, Shenzhen, China) are shown in Table 1 below.

The sensing probe was immersed in the beaker containing various NaCl concentration solutions while keeping it upright. The beaker had the capability to circulate these RI solutions. Prior to and after measuring each RI sample, the sensing probe was adequately soaked and rinsed with deionized water. As a result, a wide range of RI from 1.3471 to 1.3692 could be achieved. A medical syringe was used to inlet and outlet numerous RI solutions within the beaker.

The fiber-optic temperature measurement system with a balloon-like structure is presented in Figure 4b, using a constant temperature and humidity chamber (Xiamen Qunlong Instrument Co., Ltd., QL-GDW-18L, Xiamen, China) to provide a constant temperature field. Meanwhile, a commercial high-precision temperature and humidity meter (Xuzhou Farah Electronic Technology Co., Ltd., S20A, Xuzhou, China) was used to monitor the relative humidity (RH) and temperature of the surrounding environment in real time. During the experiment, the RH was always maintained at 50%, and the sensor was heated according to a temperature gradient of 1 °C. The corresponding transmission spectrum information was recorded when the spectrum was stable.

## 4. Results and Discussion

### 4.1. Balloon-like Fiber Sensor Fabrication

**Figure 5:**
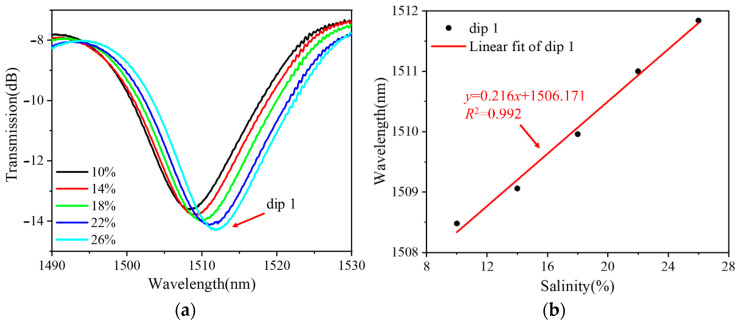
RI measurements with 10 mm sensitive length. (**a**) Transmission spectral evolution; (**b**) linear fitting curve.

**Figure 6:**
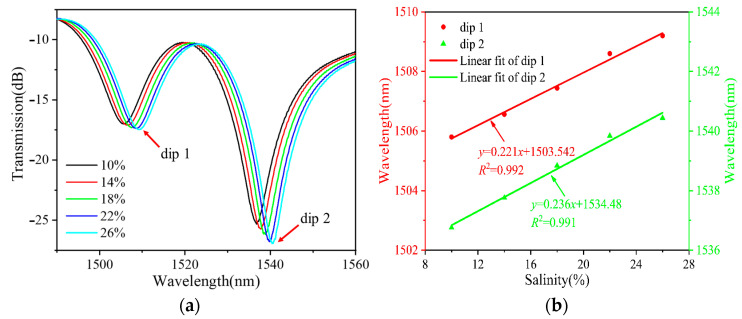
RI measurements with 15 mm sensitive length. (**a**) Transmission spectral evolution; (**b**) linear fitting curve.

**Figure 7:**
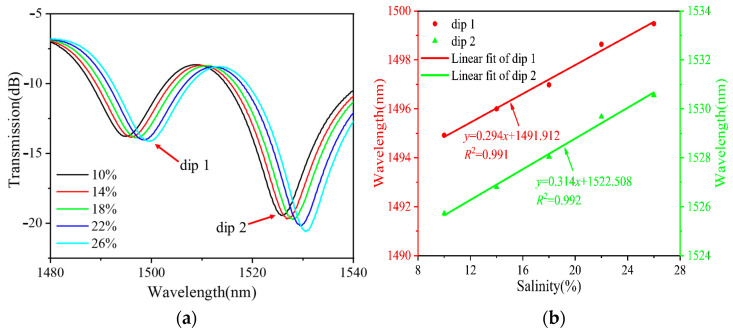
RI measurements with 20 mm sensitive length. (**a**) Transmission spectral evolution; (**b**) linear fitting curve.

Figure 5, Figure 6 and Figure 7 show the RI measurements of the balloon-like fiber interferometer for sensitive lengths of 10 mm, 15 mm, and 20 mm, respectively. Figure 5a, Figure 6a and Figure 7a depict the evolution of the transmission spectra in the range of 10% to 26% NaCl solutions with a concentration gradient of 4%. When the concentration of NaCl solutions increases, the balloon-like fiber interferometer shows a red-shift phenomenon at different sensitive lengths. This shift occurs due to the elevation of RI in the cladding modes as a result of increasing NaCl solution concentration. Consequently, this alteration influences the effective RI difference between the core mode and cladding modes, contributing to the observed red shift in the transmission spectra, and corroborating previous theoretical analyses. Taking the concentration change in NaCl solutions as the horizontal coordinate and the trough position of the transmission peak as the vertical coordinate, the linear fitting curves of the resonant wavelengths at different concentrations of NaCl solutions are plotted, as shown in Figure 5b, Figure 6b and Figure 7b. The resonant wavelength of the balloon-like fiber interference structure increases linearly when the concentration of NaCl solutions increases from 10% to 26%. The salinity sensitivity was 0.216 nm/% with a linear regression coefficient of 0.992 when the sensitive length was 10 mm. When the sensitive length was 15 mm, 0.221 nm/% and 0.236 nm/% with linear regression coefficients of 0.992 and 0.991 were obtained. A total of 0.294 nm/% and 0.314 nm/% with linear regression coefficients of 0.991 and 0.992 were obtained when the sensitive length was 20 mm, respectively.

According to the experimental results, it can be found that the sensitivity of the RI measurement increased gradually with the increase in the sensitive length (the sensitive length range was 10 mm~20 mm), and the sensitivity of salinity was the highest at 0.314 nm/% (converted to RI, it corresponded to a sensitivity of 212.88 nm/RIU). Therefore, the sensitive length of 20 mm was chosen for the subsequent ethanol filling process.

### 4.2. Temperature Sensitivity with Ethanol Solution

**Figure 8:**
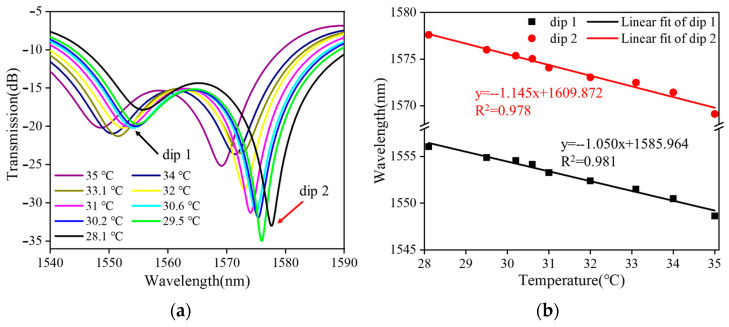
Temperature measurements of filled ethanol solution. (**a**) Transmission spectral evolution; (**b**) linear fitting curve.

Figure 8 shows the temperature measurements of a balloon-like fiber interferometer with a sensitive length of 20 mm filled with an ethanol solution. Figure 8a depicts the evolution of the transmission spectra of the sensor over the temperature range from 28 °C to 35 °C with a temperature gradient of 1 °C. When the temperature increases, the transmission spectrum of the balloon-like fiber interferometer shows a blue shift, which is due to the fact that when the temperature increases, the RI of the ethanol solution decreases and the RI of the cladding modes decreases [41,42], which alters the effective RI difference between the core mode and the cladding modes, contributing to the blue shift in the transmission spectra, which is in agreement with the previous theoretical analysis. Taking the temperature change as the horizontal coordinate and the valley position of the transmission peak as the vertical coordinate, the linear fitting curves of the resonant wavelength at different temperatures are plotted, as shown in Figure 8b. The resonant wavelength of the balloon-like fiber interference structure decreases linearly when the temperature range increases from 28 °C to 35 °C. The temperature sensitivities are −1.130 nm/°C and −1.145 nm/°C with linear regression coefficients of 0.981 and 0.978, respectively.

**Figure 9:**
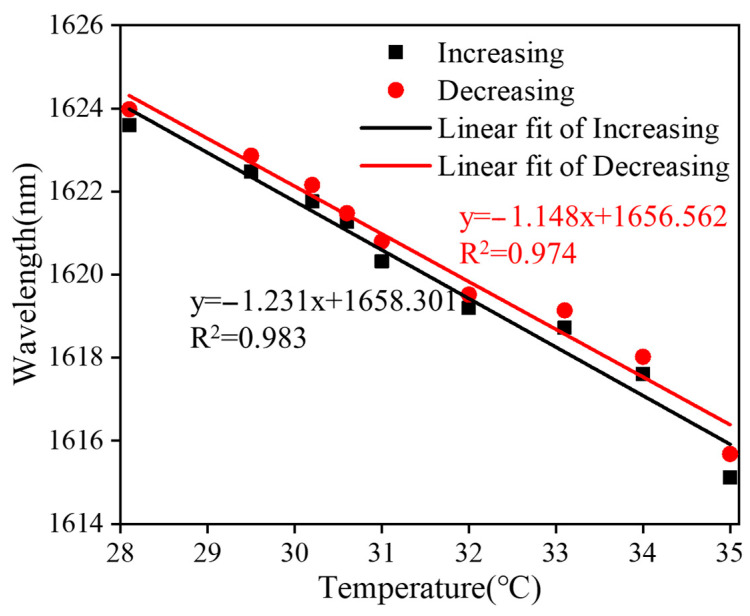
Linear fitting curves of dip 2 shifts against temperature variation.

In order to test the repeatability of the proposed FOTS, an inverse measurement cycle is applied with the temperature decreasing from 35 °C to 28 °C. When the temperature decreases, the evolution of the resonant dip feature proceeds in the opposite direction to the temperature increase process. The results of the wavelength shift fitting of dip 2 during the temperature increase and decrease are plotted in Figure 9. It can be seen that the linear regression coefficients of these two features are 0.978 and 0.981, and the temperature sensitivities are −1.145 nm/°C and −1.130 nm/°C for temperature increase and decrease processes, respectively. These findings, combined with the evolution of transmission spectral features and determined sensitivities, demonstrate the excellent repeatability offered by the proposed FOTS. The minute dissimilarities between the results obtained from the temperature increase and decrease scenarios contribute to this conclusion. Moreover, considering the 20 pm resolution of OSA utilized in the experiment, it is estimated that the FOTS can achieve a temperature resolution of 0.017 °C. It is worth noting that the interval between the resonant wavelength dip 1 and the resonant wavelength dip 2 is about 21 nm, that is, the maximum change in resonant wavelength is 21 nm, so the maximum change in temperature detected by the balloon-like structure is 21 nm/1.145 nm/°C=18.3 °C, and so when the temperature change exceeds 18.3 °C, the resonance spectrum shift is more than one period, and it will not be able to identify the temperature by OSA.

### 4.3. Temperature Sensitivity without Ethanol Solution

**Figure 10:**
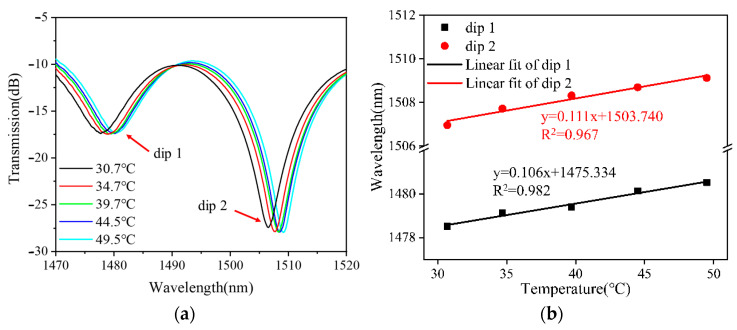
Temperature measurements of filled ethanol solution. (**a**) Transmission spectral evolution; (**b**) linear fitting curve.

Figure 10 shows temperature measurements without ethanol solution for a balloon-like fiber interferometer. Figure 10a depicts the evolution of the transmission spectra of the sensor in the temperature range from 30 °C to 50 °C with a temperature gradient of 5 °C. When the temperature increases, the transmission spectrum of the balloon-like fiber interferometer shows a red shift, which is due to the fact that when the temperature increases, the RI of the cladding modes increases due to thermo-optic and thermal expansion effects, which changes the effective RI difference between the core mode and the cladding modes, contributing to a red shift in the transmission spectra [43]. The linear fitting curves of the resonant wavelength at different temperatures are plotted with the temperature change as the horizontal coordinate and the trough position of the transmission peak as the vertical coordinate, as shown in Figure 10b. The resonant wavelength of the balloon-like fiber interference structure decreases linearly when the temperature range increases from 30 °C to 50 °C. The temperature sensitivities are 0.106 nm/°C and 0.111 nm/°C with linear regression coefficients of 0.982 and 0.967, respectively.

**Figure 11:**
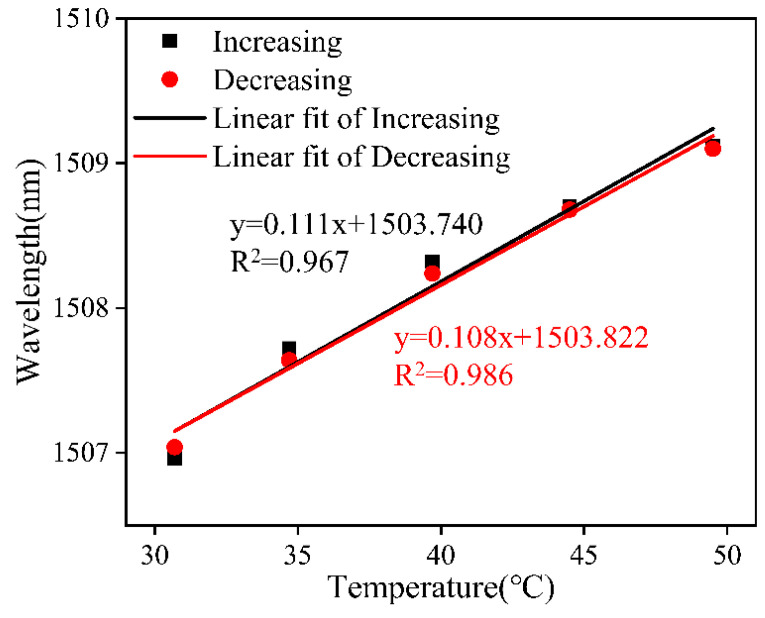
Linear fitting curves of dip 2 shifts against temperature variation.

Similarly, the FOTS unfilled with ethanol solution was subjected to a temperature increase and decrease process test to verify its repeatability. When the temperature was decreased, the resonant dip feature evolved in the opposite direction to the temperature increase process. The results of the wavelength shift fitting of dip 2 during temperature increase and decrease are plotted in Figure 11. It can be seen that the linear regression coefficients of these two features are 0.967 and 0.986, and the temperature sensitivities are 0.111 nm/°C and 0.108 nm/°C for the temperature increase and decrease, respectively. Based on the experimental results, it can be demonstrated that the unfilled ethanol fiber-optic temperature sensors are equally reproducible in both the temperature increase and decrease scenarios.

**Figure 12:**
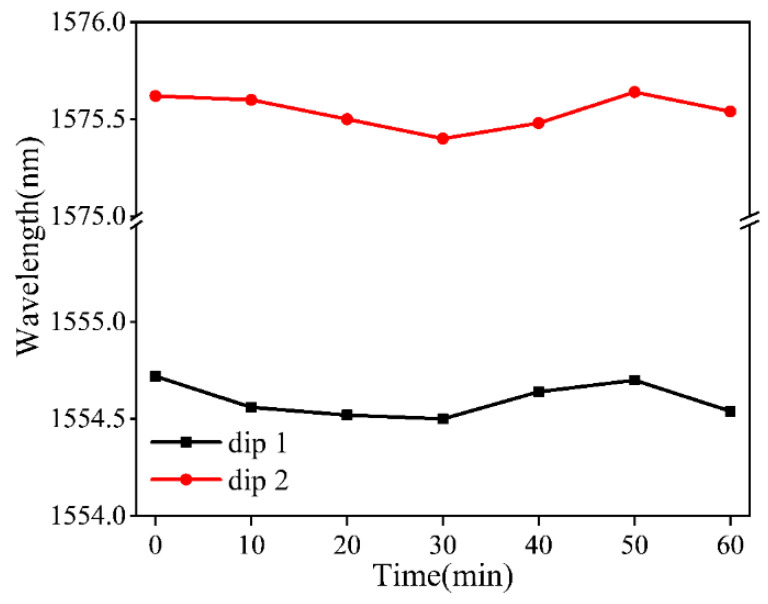
Stability response of proposed temperature sensor.

Combining the above experimental results, it can be found that the temperature detection capability of the balloon-like fiber interferometer is significantly enhanced due to the high thermo-optic coefficient of the ethanol solution. Furthermore, in practical applications, stability plays a crucial role. To assess the stability of the interferometer, the sensor was fixed in a temperature-controlled box for 60 min, with the temperature set to 30 °C, and the resonance wavelengths were recorded every 10 min. The changes in dip 1 and dip 2 are depicted in Figure 12. The stability response curves show an almost linear response, and the drift at the dip wavelength position may be owing to small variations during the experiment due to temperature instability.

To further evaluate the performance of the proposed FOTS, Table 2 shows the comparison with other fiber-optic temperature sensors with different structures. Remarkably, the proposed sensor exhibits strong competitiveness in temperature measurement compared to other reported works, while the temperature resolution of 0.017 °C is in the leading position. In addition to the direct advantages of high sensitivity and excellent resolution, the proposed FOTS also has the superiorities of simple structure and easy fabrication, which can effectively save costs. Notably, when the structure is actually used for detection, a photodetector can be used instead of a costly OSA, and a narrower band light source can be used instead of the current light source (1250–1650 nm). Combined with our previous calibration experimental results, the wavelength range of the light source only needs to include 1560–1580 nm to achieve a high-temperature sensitivity of 1.145 nm/°C and a high-temperature resolution of 0.017 °C in the range of 28–35 °C. The sensor is anticipated to have wide-ranging applications in high-precision temperature measurements, particularly within the realms of biological and medical diagnostics.

## 5. Conclusions

In this paper, a highly sensitive balloon-like FOTS filled with an ethanol solution as the sensing layer is proposed and validated for temperature measurement. Through bending an SMF stripped of the original coating layer into a balloon shape by a section of stainless steel tube and nested in a Teflon tube filled with an ethanol solution, a modal interferometer formed composed of core and cladding modes was established at a certain bending radius. In order to select the optimal sensitive length and optimize the sensor performance, combined with the size of the balloon-like structure, the sensitivity of three identical structures for RI detection was tested with the same bending radius under the sensitive length of 10 mm, 15 mm, and 20 mm, respectively, and the optimal 20 mm sensitive length for the ethanol solution was obtained. In experimental trials, the proposed sensor, which has a balloon-like structure with a bending radius of about 4 mm and a sensitive length of 20 mm, demonstrated an excellent temperature sensitivity of −1.145 nm/°C coupled with a resolution of 0.017 °C within the temperature range of 28~35 °C. The sensor proposed in this paper is easy to fabricate, low-cost, robust, and reproducible in temperature measurements, and exhibits a good linear response during temperature measurement, which provides a promising method for the high-precision detection of temperature.

## Figures and Tables

**Table 1:** RI of different NaCl concentrations.

Concentration	10%	14%	18%	22%	26%
RI	1.3471	1.3530	1.3585	1.3637	1.3692

**Table 2:** Performance of different types of fiber-optical temperature sensors.

Refs	Fiber Structure	Sensitivity	Resolution (°C)
[1]	FBG	40.4 pm/°C	1
[2]	LPG	46 pm/°C	0.3
[6]	Multicore fiber	12 pm/°C	/
[7]	Parallel double-FPIs	153.8 pm/°C	1.54
[16]	Core-offset fiber	46.2 pm/°C	0.43
[27]	SMF with an absorption layer	0.012 dB/°C	less than 1
[28]	U-shaped fiber with double coating	0.023 dB/°C	0.5
[30]	Jacket-stripped cladding-thinned SMF	0.212 nm/°C	/
[31]	Balloon-like SMF	418 pm/°C	0.173
This work	Balloon-like SMF with ethanol solution	−1.145 nm/°C	0.017

## Data Availability

The data are not publicly available due to the confidentiality and non-disclosure agreement with the funders.
